# Multidrug-resistant Mycobacterium tuberculosis: molecular perspectives.

**DOI:** 10.3201/eid0402.980207

**Published:** 1998

**Authors:** A Rattan, A Kalia, N Ahmad

**Affiliations:** Department of Microbiology, All India Institute of Medical Sciences, Ansari Nagar, New Delhi, India. rattan@medinst.ernet.in

## Abstract

Multidrug-resistant strains of Mycobacterium tuberculosis seriously threaten tuberculosis (TB) control and prevention efforts. Molecular studies of the mechanism of action of antitubercular drugs have elucidated the genetic basis of drug resistance in M. tuberculosis. Drug resistance in M. tuberculosis is attributed primarily to the accumulation of mutations in the drug target genes; these mutations lead either to an altered target (e.g., RNA polymerase and catalase-peroxidase in rifampicin and isoniazid resistance, respectively) or to a change in titration of the drug (e.g., InhA in isoniazid resistance). Development of specific mechanism-based inhibitors and techniques to rapidly detect multidrug resistance will require further studies addressing the drug and drug-target interaction.


### Multidrug-Resistant *Mycobacterium tuberculosis*: Molecular Perspectives

**Ashok Rattan, Awdhesh Kalia, and Nishat Ahmad**
All India Institute of Medical Sciences, Ansari Nagar, New Delhi, India

Multidrug-resistant strains of *Mycobacterium tuberculosis* seriously threaten tuberculosis (TB) control and prevention efforts. Molecular studies of the mechanism of action of antitubercular drugs have elucidated the genetic basis of drug resistance in *M. tuberculosis.* Drug resistance in *M. tuberculosis* is attributed primarily to the accumulation of mutations in the drug target genes; these mutations lead either to an altered target (e.g., RNA polymerase and catalase-peroxidase in rifampicin and isoniazid resistance, respectively) or to a change in titration of the drug (e.g., InhA in isoniazid resistance). Development of specific mechanism–based inhibitors and techniques to rapidly detect multidrug resistance will require further studies addressing the drug and drug-target interaction.

In the last decade, tuberculosis (TB) has reemerged as one of the leading causes of death (nearly 3 million deaths annually) (1). The estimated 8.8 million new cases every year correspond to 52,000 deaths per week or more than 7,000 each day, which translates into more than 1,000 new cases every hour, every day (2,3). These death rates, however, only partially depict the global TB threat; more than 80% of TB patients are in the economically productive age of 15 to 49 years. The emergence of AIDS and decline of socioeconomic standards contribute to the disease's resurgence in industrialized countries (4). In most developing countries, although the disease has always been endemic, its severity has increased because of the global HIV pandemic and extensive social restructuring due to rapid industrialization and conflicts. A major public health problem worldwide, TB is now a global emergency (Figure 1).

Short-course chemotherapy forms the backbone of antitubercular chemotherapy (5). Proper prescriptions and patient compliance almost always cure. In fact, TB incidence was steadily declining in most industrialized countries, until the trend was reversed (6). Further contributing to the increased death rate is the emergence of new strains of *M. tuberculosis* resistant to some or all current antitubercular drugs. The resistance is attributed primarily to improper prescriptions or patient noncompliance and is often a corollary to HIV infection (7-9). Multidrug-resistant TB (MDRTB), associated with high death rates of 50% to 80%, spans a relatively short time (4 to 16 weeks) from

Address for Correspondence: Ashok Rattan or Awdhesh Kalia, TB and STD Section, Department of Microbiology, All India Institute of Medical Sciences, Ansari Nagar, New Delhi 110 029, India; fax: 91-11-686-2663; e-mail: rattan@medinst.ernet.in or awdhesh@aiims.ernet.in.

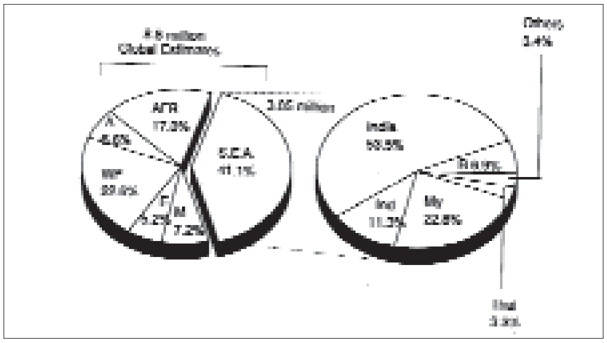

Figure 1. Global incidence of tuberculosis. Of the estimated 8.8 million cases worldwide, more than 40% of the cases are in Southeast Asia; India has approximately 53.3% of those cases. A, Americas; Afr, Africa; WP, Western Pacific; E, Europe; M, Eastern Mediterranean; and SEA, Southeast Asia; Ind, Indonesia; B, Bangladesh; Thai, Thailand; My, Myanmar. *Others include Bhutan, 0.05%; Nepal, 1.2%; Maldives, 0.001%; Sri Lanka, 1%; DPR Korea, 1.2%. (Data from reference 2).

diagnosis to death (10). Delayed recognition of drug resistance, which results in delayed initiation of effective therapy, is one of the major factors contributing to MDRTB outbreaks, especially in health-care facilities (11,12). In most countries, MDRTB has increased in incidence and interferes with TB control programs, particularly in developing countries, where prevalence rates are as high as 48% (13,14). The high infection and death rates pose an urgent challenge to rapidly detect cases.

In the past few years, genetic and molecular insights have unraveled the mechanisms involved in the acquisition of drug resistance by *Mycobacterium tuberculosis* (MTB), concomitant with the development of various molecular strategies to rapidly detect MDRTB. In this review, we examine the status of the mechanisms of resistance to antitubercular drugs.

### MDRTB and the Mechanisms of Resistance

Currently TB is treated with an initial intensive 2-month regime comprising multiple antibiotics—rifampicin (RIF), isoniazid (INH), pyrazinamide (PZA), and ethambutol (EMB) or streptomycin (SM)—to ensure that mutants resistant to a single drug do not emerge (15). The next 4 months, only RIF and INH are administered to eliminate any persisting tubercle bacilli. INH and RIF, the two most potent antituberculous drugs, kill more than 99% of tubercule bacilli within 2 months of initiation of therapy (16,17). Along with these two drugs, PZA, with a high sterilizing effect, appears to act on semidormant bacilli not affected by any other antitubercular drugs (18). Using these drugs in conjunction with each other reduces antitubercular therapy from 18 months to 6 months. Therefore, the emergence of strains resistant to either of these drugs causes major concern, as it leaves only drugs that are far less effective, have more toxic side effects, and result in higher death rates, especially among HIV-infected persons.

The phrase “MDR state” in mycobacteriology refers to simultaneous resistance to at least RIF and INH (19) (with or without resistance to other drugs). Genetic and molecular analysis of drug resistance in MTB suggests that resistance is usually acquired by the bacilli either by alteration of the drug target through mutation (20) or by titration of the drug through overproduction of the target (21). MDRTB results primarily from accumulation of mutations in individual drug target genes (Table). The probability of resistance is very high for less effective antitubercular drugs such as thiacetazone, ethionamide, capreomycin, cycloserine, and viomycin ($10^{-3}$); intermediate for drugs such as INH, SM, EMB, kanamycin, and p-amino salicylic acid ($10^{-6}$); and lowest for RIF ($10^{-8}$) (22,23). Consequently, the probability of a mutation is directly proportional to the bacterial load. A bacillary load of $10^9$ will contain several mutants resistant to any one antitubercular drug (24). Because the mutations conferring drug resistance are chromosomal, the likelihood of a mutant being simultaneously resistant to two or more drugs is the product of individual probabilities; thus the probability of MDR is multiplicative. Resistance to a drug does not confer any selective advantage to the bacterium unless it is exposed to that drug (19). Under such circumstances, the sensitive strains are killed

Table. Gene loci involved in conferring drug-resistance in *Mycobacterium tuberculosis*

| Drug | Gene | Product | Reported frequency in resistant strains[a] (%) | Reference |
|---|---|---|---|---|
| Rifampicin | *rpo*B | B-subunit of RNA polymerase | >95 | 45-48,68-71 |
| Isoniazid | *kat*G | Catalase-peroxidase | 60-70 | 39-48 |
| | *oxy*R-*ahp*C | Alky hydro-reductase | ~20 | 36 |
| INH-Ethionamide | *inh*A | Enoyl-ACP reductase | <10 | 46-48 |
| Streptomycin | *rps*L | Ribosomal protein S12 | 60 | 46-48 |
| | *rrs* | 16s rRNA | <10 | 113-117 |
| Fluoroquinolone | *gyr*A | DNA gyrase | >90 | 107 |
| Pyrazinamide | *pnc*A | Amidase | 70-100 | 92-94 |
| Ethambutol | *emb*CAB | EmbCAB | 69 | 88 |

[a]Mutation frequencies are as determined by sequencing and polymerase chain reaction-single strand conformational polymorphism (PCR-SSCP) analysis.

and the drug-resistant mutants flourish. When the patient is exposed to a second course of drug therapy with yet another drug, mutants resistant to the new drug are selected, and the patient may eventually have bacilli resistant to two or more drugs. Serial selection of drug resistance, thus, is the predominant mechanism for the development of MDR strains; the patients with MDR strains constitute a pool of chronic infections, which propagate primary MDR resistance. In addition to accumulation of mutations in the individual drug target genes, the permeability barrier imposed by the MTB cell wall can also contribute to the development of low-level drug resistance. Studies addressing resistance to SM have found evidence of such a two-step mechanism for the development of drug resistance (119,120).

### Resistance to INH

INH (isonicotinic acid hydrazide, 4-pyridinecarboxylic acid hydrazide), highly active against the MTB complex (*M. tuberculosis, M. bovis, M. africanum*, and *M. microti*), has very low MICs (0.02 µg/ml to 0.06 µg/ml) (25). The mechanism of action of INH, as well as mechanisms conferring INH resistance, are complex and not completely understood (Figure 2). However, evidence suggests that INH inhibits the biosynthesis of cell wall mycolic acids (long-chain α-branched ß-hydroxylated fatty acids), thereby making the mycobacteria susceptible to reactive oxygen radicals and other environmental factors. Activation of INH to an unstable electrophilic intermediate requires the enzyme catalase-peroxidase (KatG, coded by *kat*G) and an electron sink (hydrogen peroxide) (26), although hydrazine formed after INH spontaneously decomposes may also mediate activation of INH (27). Nevertheless, KatG is the only enzyme capable of activating INH, and consequently, KatG mutant MTB strains are invariably INH resistant.

Early studies by Middlebrook demonstrated that INH resistance was associated with loss of catalase activity (28). Genetic studies demonstrated that transformation of INH-resistant *M. smegmatis* and MTB strains with a functional KatG restored INH susceptibility and put forth the hypothesis that *kat*G deletion may cause INH resistance in MTB (29,30). However, in the absence of a peroxide-inducible genetic response, mediated in most bacteria by the transcription factor OxyR (31), KatG is the only peroxide-inducible MTB protein (32). Consequently, MTB

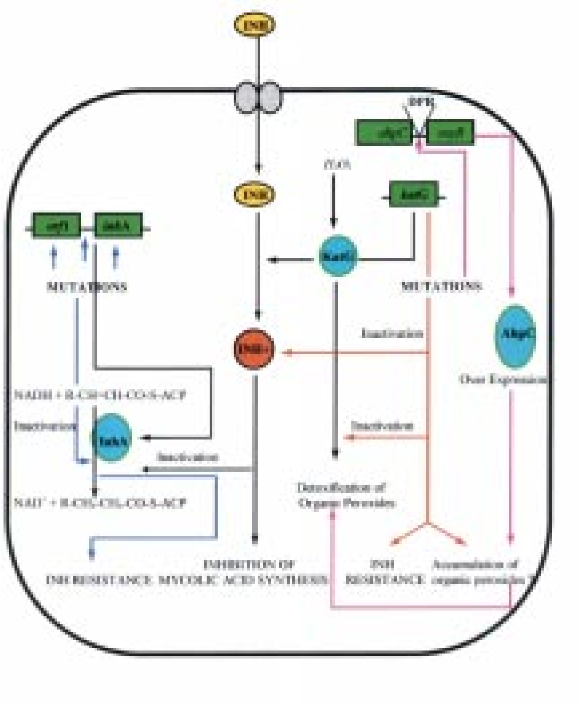

Figure 2. Mechanism of action of isoniazid (INH); acquisition of resistance and combating oxidative stress. DPR, divergent promoter region.

resistance to INH is paradoxical; it has to sacrifice KatG function. MTB's ability to adapt to the loss of KatG function and combat organic peroxides is remarkable. Studies conducted by Sherman et al. demonstrated that all KatG mutant MTB strains overexpressed a 22-kD protein at levels significantly higher than INH-sensitive strains (33). Sequence analysis confirmed that this protein was similar to the earlier reported MTB AhpC protein. AhpC can detoxify organic peroxides and is homologous to other bacterial and eukaryotic proteins with alkyl hydroperoxidase and thioredoxin-dependent peroxidase activities (34,35). The 5' regions (39 to 81 bp upstream from the *ahp*C start codon) of each AhpC-upregulated (and *kat*G mutant) isolate contained mutations that could increase promoter activity; it was proposed that compensatory mutations in the *ahp*C promoters were selected in *kat*G mutant strains to combat oxidative stress (33). Subsequent studies using immunoblotting experiments demonstrated the consistency of AhpC upregulation among clinical isolates with complete deletion of *kat*G (36,37). *kat*G mutant isolates with variable residual KatG

activity did not have this strict linear relationship. Characterization of the *oxy*R-*ahp*C region further demonstrated that mutations responsible for AhpC upregulation occurred at low frequencies and were primarily G>C to A>T transitions localized in the *oxy*R-*ahp*C intervening region (36). Although the sequence alterations in the *oxy*R-*ahp*C region were predominantly restricted to INH-resistant isolates, not all alterations detectably increased the AhpC levels. The apparent rarity of AhpC upregulation among INH-resistant and *kat*G mutant isolates could be attributed partially to the rare occurrence of MTB strains with complete *kat*G deletion (38-41,44). Alternatively, among *kat*G mutant isolates, selection of AhpC upregulatory mutations may be subject to the selective pressure exerted by residual catalase-peroxidase activity (36). However, AhpC upregulation was not observed among MTB isolates with *kat*G315 codon mutations, which reportedly lead to more than a 20-fold decrease in KatG activity and confer high MICs against INH (>90 µg/ml) (42, 43). This inconsistency and rarity of AhpC upregulation among *kat*G mutant INH-resistant isolates indicates a more complex relationship between the two and underlines the need for in-depth studies to determine precisely the conditions regulating AhpC expression.

Clinical studies to validate the paradigm of *kat*G deletions and INH resistance showed that complete deletion rarely occurred (38-41). We constructed a 35-mer oligonucleotide probe specific for *kat*G gene. Southern hybridization demonstrated the presence of *kat*G in all INH-resistant isolates, precluding complete deletion of *kat*G gene as a dominant mechanism for INH resistance (44). Previous studies using polymerase chain reaction (PCR) amplification had also established these findings; sequence analysis of *kat*G from INH-resistant strains showed randomly distributed mutations, including point mutations and deletions and insertions of up to 1 to 3 bases (38-41). These mutations could disrupt the *kat*G gene, leading to the production of an inactive gene product or a gene product with compromised peroxidative activity. PCR amplification of the *kat*G gene followed by single strand conformational polymorphism (SSCP) detected mobility shifts supporting the presence of these mutations and thereby INH resistance.

Our analysis of the *kat*G gene by PCR-SSCP resulted in the amplification of the 237 bp fragment of the *kat*G gene and demonstrated a 67.3% (n = 19) correlation between mutations in the *kat*G gene and INH resistance (45). The results were consistent with those from earlier studies indicating that *kat*G gene mutations had a correlation rate of less than 60% to 70% with INH resistance (46-48). Sequence analysis of INH-resistant strains demonstrating altered SSCP patterns showed that the most common mutation was G>T transversion in codon 463 (42). In this G>T change, Leu is substituted for Arg, and the restriction site for *Nci*I and *Msp*I is lost (40). Polymorphism in the *kat*G locus can then be easily detected by restriction digestion. Recent kinetic and spectroscopic studies have demonstrated striking similarities between KatG from wild-type strains and the R463L mutant isolates (49). Both enzymes had similar visible and electron-paramagnetic-resonance spectra and similar ability to oxidize INH and inactivate InhA. Further, when the INH-resistant *kat*G-defective strains of *M. smegmatis* with wild-type *kat*G or the R463L *kat*G were transformed, INH susceptibility was restored to about the same extent (50). These similarities do not support the contention that the R463L mutation of *kat*G allows discrimination against INH as a substrate and thereby confers resistance to INH. Although the exact role of the R463L mutation of *kat*G requires further scrutiny, this mutation may be a frequent polymorphism and may not affect INH susceptibility.

Other common mutations resulting in an attenuated KatG have been identified primarily as missense mutations that result in single amino acid substitutions (46-48). While the data point towards mutations in the *kat*G gene as the dominant mechanism for INH resistance, they also point to other factors that could mediate MTB acquisition of resistance to INH.

Mutations in the *oxy*R regulon, from which *Ahp*C is divergently transcribed, could explain the acquisition of INH resistance in the remaining INH-resistant isolates (33,51). OxyR confers high-level intrinsic resistance to INH in *Escherichia coli* and *Salmonella* Typhimurium; mutations in the *oxy*R or *Ahp*C restore INH susceptibility in these species (51). The MTB *oxy*R regulon is much smaller than in *M. leprae* and other mycobacteria—because of two important deletions of 29 bp and 372 bp (32,52). In addition to these deletions, the *oxy*R regulon carries many frame shift mutations, which result in low expression of this regulon and eventually lead to low-level expression of AhpC (consistent with the finding of low-level expression of AhpC in INH-sensitive strains vs. INH-resistant strains) (33). A related member of the genus resistant to INH, *M. leprae*, however, has a complete *oxy*R-*ahp*C region that is transcriptionally fully active and may play a role in the detoxification of active INH intermediates (52). By analogy, therefore, the loss of the OxyR function, in conjunction with its putative effects on *ahp*C expression, could explain the exquisite specificity of INH for the MTB complex. However, evidence from recent studies does not indicate a direct role for *oxy*R or the *ahp*C genes in determining susceptibility to INH (36,37). Polymorphisms in *oxy*R do not have any preferential predisposition and exist among both INH-resistant and -susceptible isolates with about the same frequency (36). The relationship of AhpC overexpression to INH resistance is more complex. Earlier observations based on transformation of *M. smegmatis* strains suggested a possible involvement of AhpC overexpression in acquiring INH resistance (53). Transformation of *M. smegmatis* isolates with multicopy constructs of *ahp*C led to almost a fivefold increase in the MIC for INH. However, an increasing body of evidence precludes any direct role of AhpC in determining INH susceptibility among MTB isolates. MTB transformants bearing multicopy constructs of *ahp*C did not demonstrate significant increase in the MIC for INH, thus any direct role for AhpC in acquisition of INH resistance was ruled out (37).

Efforts to determine the factors involved in resistance to INH led to the discovery of the *inh*A locus, which was proposed as the primary target for coresistance to INH and ethionamide (54). This locus is composed of two open reading frames (ORFs), designated *orf*1 and *inh*A, separated by a 21-bp noncoding region. InhA, an enoyl-ACP reductase (55), more than 40% homologous to the EnvM protein, catalyzes an early step in fatty acid synthesis among enterobacteria. Like EnvM, InhA activity is also thought to use NAD(H) as cofactor. INH susceptibility could result from incorporation of iso-NAD, which is formed as a consequence of the action of KatG on INH, and thus hinders the enzymatic activity of InhA and blocking fatty acid synthesis (56). A T>G transversion, observed in few of the resistant strains, at position 280 in the *inh*A gene, results in the ser94 to ala94 replacement (54). This replacement, thought to alter the binding affinity of InhA to NAD(H), ultimately results in INH resistance (57). Alternatively, because of mutations in the putative promoter region, hyperexpression of InhA could result in INH resistance.

Studies conducted in clinical settings to provide corroborating evidence of mutations in the *inh*A locus and INH resistance have shown approximately 10% correlation (46-48). Analysis of 37 INH-resistant isolates by Kapur et al. demonstrated no ser94-ala94 substitution in the resistant isolates. Only one isolate had a missense mutation: ATC>ACC at position 47, resulting in substitution of Ile16 by Thr16. Morris et al. also demonstrated the lack of mutations in the *inh*A gene among 42 INH-resistant MTB isolates. However, five of the INH-resistant isolates showed single nucleotide mutations in the putative *inh*A regulatory region upstream of *orf*1.

Subsequent biochemical characterization of InhA function demonstrated that it catalyzed the reduction of 2-trans-octenoyl-acyl carrier protein and also that protein of enoyl CoA esters (58-590, thereby acting at the final step in chain elongation in fatty acid synthesis (58). This observation contradicted earlier biochemical evidence suggesting that an enzyme involved in the synthesis of an unsaturated 24-carbon fatty acid was the target for activated INH (60,61). Thus, the targets identified biochemically and by complementation of *M. smegmatis* are different. Lipid pulse labeling experiments demonstrated that the lipid biosynthetic response of *M. smegmatis* and MTB after exposure with INH were different (62), indicating a different mechanism of action for the INH intermediate in the two species. Transformation of *M. smegmatis* with single-copy alleles of mutant *inh*A loci did not result in significant resistance to INH, indicating the presence of a different promoter in *M. smegmatis*. Further, the inability of multicopy vector constructs bearing the *inhA* gene to significantly increase the MIC for INH provided substantiating evidence for the limited involvement of this locus in mediating INH resistance among MTB isolates. These data, along with clinical evidence, preclude the likelihood that *inh*A is the primary target for the activated form of INH.

Functional characterization of *inh*A mutations, occurring with *kat*G mutations (as observed in isolates with very high MICs) (46) in relation to lipid metabolism of INH-resistant isolates, could perhaps resolve this discrepancy and delineate the roles of the respective loci in the mechanism of action of INH and subsequent acquisition of drug resistance.

In summary, mutations in the *kat*G and the *inh*A genes are associated with approximately 70% to 80% of INH-resistant MTB isolates; molecular mechanisms operating in the remaining isolates are still unknown. The role of the MTB cell wall as an important permeability barrier needs to be explored in greater detail, particularly with reference to INH resistance (56).

### Resistance to RIF

RIF, first introduced in 1972 as an antitubercular drug, is extremely effective against MTB. It has MICs of 0.1 µg to 0.2 µg (16,63). Because of its high bactericidal action, RIF, along with INH, forms the backbone of short-course chemotherapy (5). Although rare, resistance to RIF is increasing because of widespread application and results in selection of mutants resistant to other components of short-course chemotherapy. In this context, resistance to RIF can be assumed to be a surrogate marker for MDRTB (19). RIF had long been believed to target the mycobacterial RNA polymerase and thereby kill the organism by interfering in the transcription process (64). Using purified RNA polymerase from *M. smegmatis*, strain mc$^{2}$155, Levin and Hatfull demonstrated that RIF specifically inhibited the elongation of full-length transcripts and had virtually no effect on the initiation of transcription (65).

RNA polymerase, a complex oligomer composed of four different subunits (α,ß,ß'and σ, encoded by *rpo*A, *rpo*B, *rpo*C, and *rpo*D, respectively), is highly conserved among bacterial species (66). Characterization of the *rpoB* gene in *E. coli* demonstrated that RIF specifically interacted with the ß subunit of RNA polymerase, thereby hindering transcription, and that mutations in the *rpo*B locus conferred conformational changes leading to defective binding of the drug and consequently resistance (67). Subsequently, the *rpo*B locus from MTB was characterized and mutations conferring the resistant trait were identified (Figure 3; 68-71). Most mutations were determined to be restricted to an 81-bp core region and are dominated by single nucleotide changes, resulting in single amino acid substitutions, although inframe deletions and insertions also occur at lower frequencies. Changes in the codons Ser531 and His526 have been documented in more than 70% of the RIF-resistant isolates. A very small number of mutations in RIF-resistant isolates do not map in this 81-bp core region; it is speculated that additional mechanisms, including RIF permeability and mutations in alternate subunits of RNA polymerase, may also be involved in conferring the resistance phenotype.

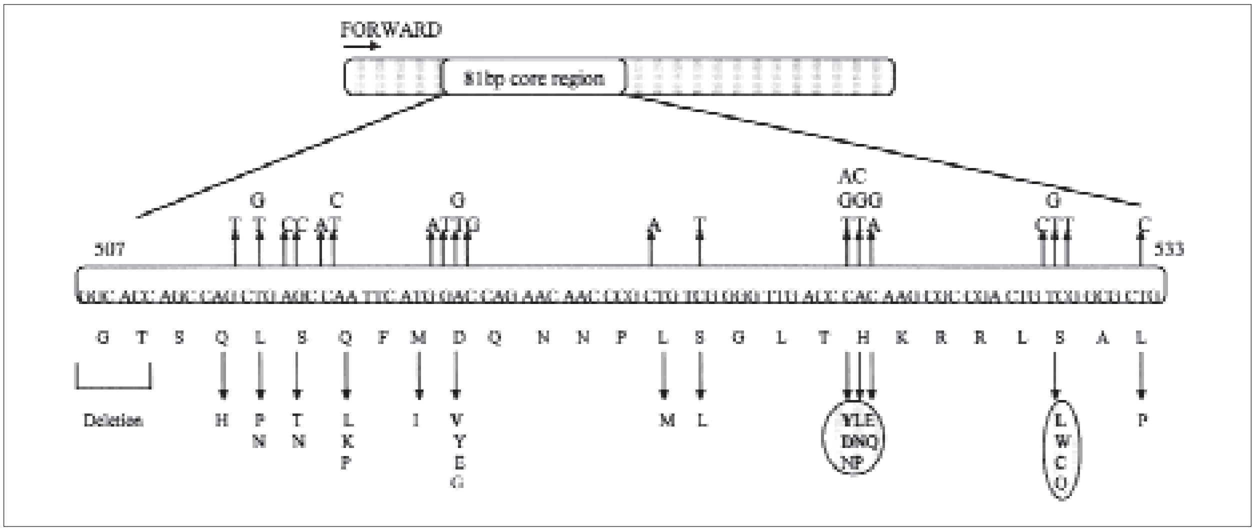

Figure 3. Single amino acid substitutions in the 81 bp core-region of the *rpo*B gene responsible for conferring rifampicin (RIF) resistance (Insertions and deletions that confer the RIF-resistance phenotype are not depicted). Amino acids are represented with single letter abbreviations. Changes in codon Ser531 and His526 account for more than 70% of the mutations with RIF resistance (depicted in shaded ellipses).

The consistency of mutations in the *rpo*B locus and the RIF- resistant phenotype (>95%) has marked clinical implications. Because it may act as a surrogate marker for MDRTB, RIF resistance has prompted development of various diagnostic tests to improve the sensitivity of mutation detection. Although automated sequencing has been unambiguously applied to characterize mutations associated with RIF resistance, a number of other techniques such as PCR-SSCP (41,45-48,121), dideoxy fingerprinting (72), heminested PCR (73), PCR heteroduplex analysis (70), and line probe hybridization (74,75) have been successfully applied to detecting these mutations. Such novel strategies to detect drug-resistant MTB isolates have been described elsewhere (76). PCR-SSCP analysis for detection of mutations responsible for conferring drug-resistance is increasingly useful. In particular, the development of nonisotopic PCR-SSCP analysis has simplified the procedure, enhancing its utility in routine laboratories (41,45). However, results obtained with SSCP analysis should be interpreted with caution as the technique only detects mutations and gives no information on the nature of associated mutation. For example, silent mutations in the *rpo*B gene have been identified that give altered mobility patterns on SSCP analysis but have no association with RIF resistance, which underlines the need for caution in interpreting results and phenotypic or genotypic correlation (77).

### Resistance to EMB

EMB [dextro-2,2'-(ethyldiimino)-di-1onol], synthetic compound with profound antimycobacterial effects (78), is a first-line anti-MTB drug with a broad spectrum of activity, unlike INH. EMB is also advocated in disseminated *M. avium* complex infections, particularly in HIV-infected persons (79). Until recently, EMB's mechanism of action and the genetic basis for resistance to it were largely obscure. Specificity of EMB for mycobacterial species, however, indicated that its target may have been involved in the construction of the outer cell wall. Synergy resulting from coadministration of EMB and other drugs gave further evidence for the involvement of EMB in obstructing the formation of cell wall. The synergistic effect was explained as a consequence of increased permeability of the mycobacterial cell wall leading to increased drug uptake (80,81). Indeed, earlier studies of Takayama and colleagues demonstrated that administration of EMB led to rapid cessation of mycolic acid transfer to the cell wall and equally rapid accumulation of trehalose mono- and di-mycolates (82,83). Mycolic acids attach to the 5'-hydroxyl groups of D-arabinose residues of arabinogalactan and form mycolyl-arabinogalactan-peptidoglycan complex in the cell wall. Disruption of the arabinogalactan synthesis inhibits the formation of this complex and may lead to increased permeability of the cell wall. Subsequently, it was demonstrated that EMB specifically inhibited arabinogalactan synthesis (84).

A breakthrough was achieved in defining the precise cellular target for EMB with the isolation and identification of ß-D-arabinofuronosyl-1-monophosphoryl decaprenol (DPA), which accumulates rapidly (less than 2 minutes) on exposure of EMB- sensitive cells to EMB (86). DPA is an arabinosyl donor; cell-free assay systems developed for DPA established that it was one of the major intermediates of arabinan synthesis. It was later shown that EMB specifically inhibited arabinosyl transfer, suggesting that arabinosyl transferase was the primary cellular target for EMB (Figure 4).

Identification of arabinosyl transferase as the primary target for EMB helped unravel the

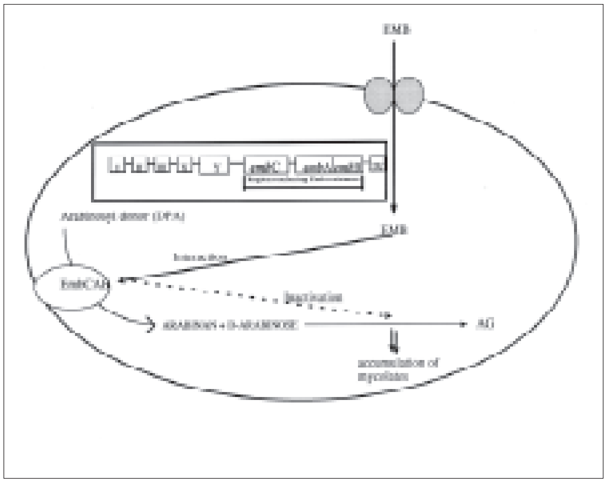

Figure 4. Mechanism of action of ethambutol (adapted from 84-88). EMB interacts with the EmbCAB proteins encoded by the *emb*C, *emb*A, and *emb*B genes, leading to inactivation of arabinogalactan synthesis. Mutations in the *emb*B locus cause alterations in EmbB, possibly leading to an altered target for EMB. Alternatively, hyperexpression of the EmbCAB proteins could lead to EMB resistance. *Inlet box*: Organization of the *emb* operon in *Mycobacterium tuberculosis* (MTB).

genetic basis for EMB resistance. Using target overexpression by a plasmid vector, Belanger et al. cloned the *emb* locus from an EMB-resistant strain of *M. avium* (86). Transformation of this *emb* locus conferred resistance to *M. smegmatis* mc[2]155 strain and also demonstrated that the level of resistance conferred depended on the copy number of the gene, which was consistent with the notion of drug resistance due to target overexpression. Site-directed mutagenesis and overlapping clone analysis localized a 9.8-kb EMB resistance locus, subsequently shown to be ubiquitous among mycobacteria. Sequence analysis of this locus revealed three complete ORFs—designated *emb*R, *emb*A, and *emb*B. The *emb*R ORF is separated by a 178 bp divergent promoter region from the *emb*A and *emb*B ORFs. Characterization of the *emb*R ORF showed that the region was strongly homologous with a family of transcriptional activators of *Streptomyces* and thus could play a role in modulating the expression of *emb*A and *emb*B. Importantly, the *emb*B ORF lacks a potential ribosome binding site and is thus translationally coupled to *emb*A, which suggests that a heterodimeric enzyme complex may be the target for EMB. Mapping studies further demonstrated that both *emb*A and *emb*B, along with the divergent promoter region, were essential to EMB resistance.

In contrast to the organization of the emb locus in *M. avium*, molecular genetic approaches applied to MTB revealed a highly conserved 14-kb region comprising three homologous ORFs designated embC, embA, and embB preceded by a predicted coding region and by orfX (which encodes a putative protein belonging to the short chain alcohol dehydrogenase family) (87). Primer extension analysis of the emb region supported the notion of its organization as an operon and further indicated the polycistronic nature of its transcripts. The emb genes are translationally coupled the absence of any untranslated intercistronic region between the emb genes so indicated). However, the presence of a secondary stem loop structure between the embA and the embB genes indicates that the embB gene in MTB could be differentially regulated. The embCAB proteins are believed to be integral membrane proteins, consistent with their role in the synthesis of various arabinan-linkage motifs of the arabinogalactan and lipoarabinomannan (86,87).

Identification of the *emb*CAB genes prompted a detailed analysis of the molecular mechanisms responsible for conferring resistance to EMB in MTB isolates. Preliminary studies documented among EMB-resistant isolates missense substitutions in the conserved *emb*B codon 306 that coded for methionine; their role in conferring resistance to EMB was confirmed by gene tranfer assays (87). Recent analysis of the *emb*CAB region has confirmed the predominance of *emb*B Met306 substitutions among EMB-resistant clinical isolates of MTB (approximately 89% among EMB-resistant isolates with single amino acid substitutions) (88). Sequence analysis of 118 clinical isolates of MTB showed five mutants of the *emb*B codon 306, all leading to substitution of Met with Val, Leu, or Ile. MTB strains with Met306Leu and Met306Val substitutions demonstrated a higher MIC for EMB (40 µg/ml) than those for organisms with Met306Ile substitutions (20 µg/ml). The *emb*B codon 306 may contain important structure-function information; structural alterations in this codon may have a detrimental effect on the interaction of EMB and EmbB, thereby resulting in a EMB-resistant phenotype.

Sequence alterations in the *emb*CAB region correlate with approximately 70% of EMB-resistant strains. Overexpression of the EmbB protein has been documented to mediate resistance in *M. smegmatis* (87), and a homologous mechanism may operate in MTB, perhaps accounting for the remaining 30% of the EMB-resistant isolates. A full understanding of the mechanisms for acquisition of EMB resistance among these isolates requires further studies.

### Resistance to PZA

PZA, a structural analog of nicotinamide, was shown to have considerable anti-MTB activity in 1952, but it became an important component of short-course chemotherapy only in the mid-1980s. PZA, active against semidormant bacilli not affected by any other drug, has strong synergy with INH and RIF and shortens the chemotherapeutic schedule for antitubercular treatment from 9 to 12 months to 6 months (15). Depending on the assay system and conditions applied, MICs of PZA vary from 8 µg/ml to 60 µg/ml. However, even at very high MICs, PZA has no significant bactericidal effect and is primarily considered a “sterilizing drug” (18). Activity of PZA is highly specific for MTB; PZA has scant or no effect on other mycobacteria, including *M. bovis*, which demonstrate high-level intrinsic resistance to PZA (89). Naturally resistant strains of *M. bovis* lack the enzyme Pzase, which hydrolyzes PZA to pyrizinoic acid, the presumed active form of PZA (90,91). PZA in this context is similar to INH; it is transported as a neutral species into the cell, where it is converted into its active form. This notion was strengthened by evidence provided by in vitro studies that demonstrated the susceptibility of PZA-resistant MTB and *M. bovis* to pyrizinoic acid. MTB Pzase has both pyrazinamidase and nicotinamidase activities (90). Using sequence information of *E. coli* nicotinamidase, Scorpio and Zhang isolated the mycobacterial *pnc*A gene, which codes for the amidase (92). Characterization of the *pnc*A gene from *M. bovis* isolates identified a single point mutation that results in the substitution of His to Asp at position 57. This substitution results in the production of an ineffective Pzase in *M. bovis* strains. Point mutations in the *pnc*A gene of PZA-resistant MTB strains were also identified. Substitution of Cys138 with Ser, Gln141 with Pro, and Asp63 with His and deletion G nucleotide at positions 162 and 288 resulted in a defective Pzase. Transformation of Pzase-resistant strains with functional construct of MTB *pnc*A gene restored susceptibility to PZA, providing further evidence that mutations in the *pnc*A gene were responsible in conferring the resistant phenotype. Subsequent characterization of the *pnc*A gene from clinical isolates of MTB confirmed these findings (93,94). Mutations including missense alterations, nucleotide insertions or deletions, and termination mutations have been found in the *pnc*A gene from PZA-resistant MTB isolates. These sequence alterations are interspersed along the entire length of the *pnc*A gene, demonstrate limited degree of clustering, and vary in frequency from 70% to 100% (93,94). The absence of correlating mutations in the *pnc*A gene from PZA- resistant MTB isolates indicates that perhaps at least one additional mechanism mediates resistance to PZA.

The cellular target for PZA, however, has not been identified, although the apparent similarity of PZA to nicotinamide suggests that enzymes involved in pyridine nucleotide biosynthesis are probable targets. Implication of the *pnc*A gene in conferring PZA-resistant phenotype has profound clinical applications. Application of PCR-SSCP for detection of mutations in the *pnc*A gene could help circumvent the difficulties in determining PZA susceptibilities (96) and rapidly discriminate between MTB and *M. bovis* (96).

### Resistance to Fluoroquinolones (FQ)

FQs as antimycobacterial agents were first described in 1984 and have primarily been used as therapeutic alternatives in MDRTB cases (97). DNA gyrase (Gyr), a member of the type II DNA topoisomerases (98), is the primary target for FQ action. Gyr introduces negative supercoils in closed circular DNA molecules and is a heterotetramer ($A_2B_2$), coded by *gyr*A and *gyr*B respectively (99,100). Quinolone sensitivity is determined by the GyrA protein, which contains the cleavage/religation activity (100), while GyrB contains the intrinsic coumarin-sensitive ATPase activity (101).

FQs, synthetic derivatives of nalidixic acid, act by inhibiting DNA supercoiling and relaxation activity of Gyr without affecting the ATPase activity (102) and enhance the rate of DNA cleavage by Gyr. Quinolone-mediated cleavage of double-stranded DNA results in a 4 bp 5' overhangs on either strand, to which GyrA subunits become attached covalently by $O^4$ phosphotyrosine bond (103). Gyr catalyzes the cutting of DNA, denaturation of the overhang, and strand separation. The exact mechanism of inhibition of Gyr activity with respect to quinolones remains unknown. However, quinolone drugs bind with a greater affinity to single-stranded DNA than double-stranded DNA and possibly do not bind to Gyr at all (104). Consequently, by binding to the single-stranded DNA, the quinolones may inhibit religation, thereby imposing an effective transcriptional block (105), culminating in cellular death. However, questions about the specific interaction of quinolones and the Gyr/DNA complex remain unsolved (106).

Cloning and expression of the MTB *gyr*A and *gyr*B genes allowed mapping of mutations that confer resistance to FQs (107). Mutations were found to be clustered in a small region in GyrA that is close, approximately 40 residues amino-terminal, in the linear amino acid sequence to the active site tyrosine, Tyr122 (*E. coli* numbering) (108). Other single amino substitutions, for residues 88 to 94, were also identified in ciprofloxacin-resistant MTB isolates (Figure 5). Because polymorphism encountered at codon 95 (Ser95>Thr95) occurred in both resistant and susceptible isolates, it may not be involved in

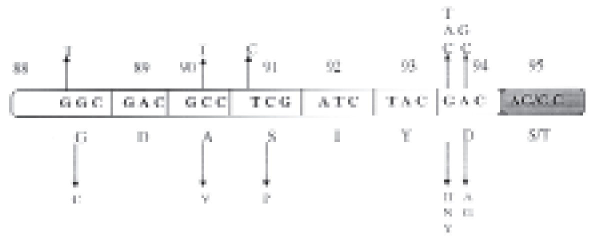

Figure 5. Single-amino acid substitutions responsible for conferring resistance to fluoroquinolones (FQ). Mutation in the Ser95 codon (shown in stippled box), observed in both FQ-sensitive and FQ-resistant isolates, rules out its role in acquisition of resistance.

acquiring the FQ-resistant phenotype. Alternative mechanisms to *gyr*A mutations, including changes in cell wall permeability and active quinolone efflux pumping, have also been proposed and could account for the low-level resistance among MTB isolates.

Newer FQ derivatives such as sparfloxacin have shown greater anti-MTB potency (MIC = 0.2 µg/ml) than ciprofloxacin and ofloxacin, giving hope for better therapeutic alternatives for MDRTB. However, FQ susceptibility in the treated patient population must be continuously monitored to prevent low-level FQ-resistant strains from acquiring additional mutations that lead to high-level resistance (109).

### Resistance to Streptomycin and Other Inhibitors Of Protein Synthesis

Various drugs exert their antibacterial effects by inhibiting the protein transitional machinery. Among these, aminoglycosides, macrolides, tetracyclines, and basic peptides like viomycin and capreomycin are active against mycobacteria (110). SM, one of the oldest drugs known to be active against MTB, disrupts the decoding of aminoacyl-tRNA and thus inhibits mRNA translation or causes inefficient translation (111). One of the most common mechanisms for acquisition of resistance to SM is acetylation of the drug by aminoglycoside-modifying enzymes (111,112). However, this mechanism is not found in MTB. Instead, resistance to SM is attributed, at least partially, to two distinct classes of mutations including point mutations in S12 ribosomal protein, encoded by *rps*L gene (113), and mutations in the *rrs* operon encoding the 16S rRNA (114).

Point mutations in the *rps*L gene result in single amino acid substitutions (114-117) that affect higher order structures of 16S rRNA and thereby confer SM resistance. Mapping of the mutations in the *rps*L gene demonstrated that they primarily affected one of the two critical lysine residues at positions 43 and 88 and led to the substitution with either arginine at 88 or arginine and threonine at position 43 (115). An SM-resistant isolate (>60 µg/ml) showed an A>G transversion at position 904 in the 16S rRNA with an additional single A>C transversion in the *rps*L gene, which resulted in the substitution of Lys-Gln at position 88 (115). Because each of the corresponding mutations in the small subunit rRNA or the ribosomal protein S12 confer the resistant phenotype in *E. coli*, these mutations mediated ribosomal drug resistance and were responsible for conferring high-level SM resistance. Mutations in the *rps*L gene accounted for more than two thirds of SM-resistant cases.

The genesis of SM resistance in some of the SM-resistant isolates is due to point mutations in the 16S rRNA. Mutations in the *rrs* locus have been mapped to two regions, the 530 loop and the 915 region. Within the 530 loop, C>T transitions at 491, 512, and 516, in addition to the A>C transversion at position 513, are consistent with the SM-resistant phenotype (114) pseudoknot formation within the MTB 16S rRNA. Base pairing between residue 524-526 (of the 530 region of the hairpin loop) and residue 504-507 (of the adjacent 510 region bulge loop) (118) results in SM resistance in clinical isolates of MTB (114). Further, G-U wobble base pairing between residues 522-501 stabilizes the pseudoknot formation and thereby confers resistance to SM. It can thus be concluded that SM resistance in MTB stems from alterations of the drug target and not by drug modification.

However, no mutations in the *rps*L and the *rrs* genes are detected in a significant number of SM-resistant isolates (46,48). Curiously, intrinsically SM-resistant strains of *M. gordonae*, *M. szulgae*, and *M. avium* do not show any alterations in the *rps*L or the *rrs* genes, suggesting a probable third factor in conferring SM resistance. Earlier studies have documented the inhibitory effect of SM on protein synthesis in vitro to the same extent as observed in wild-type MTB strains. The same inhibitory effect was not observed on whole cells, suggesting the probable role of cell wall permeability barrier in conferring SM resistance (119). More recently, it has been demonstrated that membrane-active substances augmented the MIC for SM in strains with alterations in the *rrs* genes, thus providing further evidence for a probable role of the MTB-permeability barrier in mediating resistance to SM (120).

### Resistance to Other Drugs

Related aminoglycosides such as kanamycin, amikacin, and paromomycin demonstrate no obvious cross-resistance to SM and thus are alternatives in cases of SM resistance. Viomycin and capreomycins are bacteriostatic agents that act by binding to the 50S ribosomal subunit and inhibit the translocation reaction (111). Although cross-resistance between viomycin and capreomycin does occur, the exact mechanism for acquisition of drug resistance is not known.

### Conclusions

Molecular insights suggest that accumulation of mutations in the individual drug target genes is the primary mechanism of MDRTB. Morris and colleagues' investigation of the molecular mechanisms of drug resistance in MDR strains found that 25 of 44 SM-resistant strains had mutations in the *rps*L gene, while five others had *rrs* gene perturbations (48). The *rpo*B gene had mutations in 28 of 29 RIF-resistant strains. Mutation in the *kat*G gene was seen in 20 of the 42 INH-resistant stains, while five had *inh*A gene mutations. Of the 20 MDRTB strains, 11 had mutations in genetic markers associated with resistance to each of these three drugs.

Similarly, Heym et al. reported that resistance to antitubercular agents in their collection of strains resulted from alterations to chromosomal genes encoding the drug targets; they excluded the possibility that MDRTB stemmed from acquisition of genes for novel resistance determinants (46). MDR appeared to result from the stepwise acquisition of new mutations in the genes for different drug targets. In all cases exactly the same mutations or combination of mutations were observed, regardless of the patient's HIV status.

Thus, the origin of MDRTB is due more to treatment difficulties, including noncompliance and administration of inadequate treatment regime, and not to the emergence of novel resistance mechanisms; this is reassuring for the future of short-course chemotherapy. Administration of directly observed combination chemotherapy (or Directly Observed Treatment Short-Course [DOTS]) appears to be the most effective way to ensure a decrease in primary resistance, acquired resistance, and relapses (3). DOTS has been successfully implemented in diverse geographic areas including Tanzania, Guinea, China, Bangladesh, New York City, and Peru, which reported more than a 90% cure rate (3). Nearly 70 countries have adapted DOTS as a part of their national TB control programs and achieve good cure rates. Successful implementation of DOTS in the coming decades requires not only a concerted effort from various funding agencies but also a strong social and political commitment. Apart from strategic interventions based on strong political will, grass-roots action will have to be strengthened mainly at the primary health-care level to check the unlimited upsurge of this preventable fatal disease. Basic research will have to be continually updated to prevent the drug-resistant strains from becoming an unmanageable clinical paradigm. DOTS currently is our only option to reverse the global TB epidemic and prevent MDRTB.

The inability to detect resistance early, however, is one of the major factors involved in the genesis and control of MDRTB; this invariably results in prolonged exposure to drugs that are virtually ineffective. One of the major consequences of unraveling the genetic basis of drug resistance in MTB is the development of various molecular strategies to rapidly detect MDRTB (76). However, the sheer multiplicity of gene loci to be investigated for diagnosis of MDRTB renders most of the approaches mentioned above as tedious and resource-intensive for a routine laboratory service program, particularly in developing countries like India, with limited resources and high disease incidence. Resistance to most anti-MTB drugs, with the exception of RIF, cannot be attributed to a single locus in substantial percentage (>90%), which is perhaps the greatest deterrent in the development of single amplification–based methods for rapid detection of resistance.

Working out the exact biochemical details of drug-drug target interaction acquires considerable attention in the era of MDRTB, because only then will more rational structure- and mechanism-based approaches to inhibitor design be possible. Clearly, a concerted global effort is required to defeat TB resurgence.

### Acknowledgments

We are indebted to Dr. Ellen Jo Baron and Prof. Eric C. Bottger for their suggestions and encouragement; Dr. James M. Musser for providing recent data on ethambutol resistance; and Swathi Arur, Nadeem Hasan, and Koninika Ray for help in preparing this manuscript.

Work in our laboratory is supported by financial grants from the Department of Biotechnology, Government of India.

Dr. Ashok Rattan is an additional professor, Department of Microbiology, All India Institute of Medical Sciences, New Delhi, India. His research focuses on drug resistance and application of cost-effective methods for surveillance of MDRTB strains.

### References

1. Bloom BR, Murray CJL. Tuberculosis: Commentary on a reemergent killer. Science 1992;257:1055-64.
2. World Health Organization. Bridging the gaps: the world health report. Geneva: The Organization; 1995.
3. World Health Organization report on TB epidemic. Global TB programme. Geneva: The Organization; 1997.
4. Barnes P, Blotch AB, Davidson BT, Snyder Jr DE. Tuberculosis in patients with immuno-deficiency virus infection. N Engl J Med 1991;324:1644-50.
5. Kochi A, Vareldzis B, Styblo K. Multi-Drug resistant tuberculosis and control. Res Microbiol 1993;144:104-10.
6. Bell RT. Tuberculosis of the 1990s: the quiet public health threat. Pa Med 1992;95:24-5.
7. Snyder DE Jr, Roper WL. The new tuberculosis. N Engl J Med 1992;326:703-5.
8. Freiden TE, Sterling T, Pablos-Mendez A, Kilburn JO, Cauthen JO, Dooley SW. The emergence of drug-resistant tuberculosis in New York city. N Engl J Med 1993;328:521-6.
9. Nosocomial transmission of multi-drug resistant tuberculosis among human immuno-deficiency virus infected patients—Florida and New York, 1988-1991. MMWR Morb Mortal Wkly Rep 1991;40:585-91.
10. Dooley SW, Jarvis WR, Martone WJ, Snyder DE Jr. Multi-Drug resistant tuberculosis [editorial]. Ann Intern Med 1992;117:257-8.
11. Edlin BR, Tokers JI, Greeko MH, Crawford JT, Williams J, Sordillo EM, et al. An outbreak of multi-drug resistant tuberculosis among hospitalized patients with the Acquired Immuno-Deficiency syndrome. N Engl J Med 1992;326:1514-21.
12. Pearson ML, Jareb JA, Freiden TR, Crawford JT, Davis BJ, Dooley SN, et al. Nosocomial transmission of multi-drug resistant tuberculosis—a risk to patients and health care workers. Ann Intern Med 1992;117:191-6.
13. Iseman MD, Sbarbaro JA. The increasing prevalence of resistance to antituberculosis chemotherapeutic agents: implications for global tuberculosis control. Curr Clin Top Infect Dis 1992;12:188-204.
14. Cohn DL, Flavia B, Raviglione MC. Drug-resistant tuberculosis: review of the worldwide situation and the WHO/IUATLD global surveillance project. Clin Infect Dis 1997;24:S121-30.
15. Initial therapy for tuberculosis in the era of multi-drug resistance: recommendations of the advisory council for the elimination of tuberculosis. MMWR Morb Mortal Wkly Rep 1993;42 (RR-7).
16. Mitchison DA. Mechanism of drug action in short-course chemotherapy. Bulletin International Union Against Tuberculosis 1985;65:30-7.
17. Iseman MD, Madsen LA. Drug-resistant tuberculosis. Clin Chest Med 1989;10:341-53.
18. Heifets LB, Lindohlm-Levy PJ. Pyrazinamide sterilizing activity in vitro against semidormant *Mycobacterium tuberculosis* populations. American Review of Respiratory Diseases 1992;145:1223-5.
19. Vareldzis BP, Grosset J, de Kantor I, Crofton J, Laszlo A, Felten M, et al. Drug-resistant tuberculosis: laboratory issues. World Health Organization recommendations. Tubercle and Lung Diseases 1994;75:1-7.
20. Spratt BG. Resistance to antibiotics mediated by target alterations. Science 1994;264:388-93.
21. Davis J. Inactivation of antibiotics and the dissemination of resistance genes. Science 1994;264:375-82.
22. Shimao T. Drug-resistance in tuberculosis control. Tubercle 1987;68(suppl):5-15.
23. Crofton J. The assessment and treatment of drug-resistance problems in tuberculosis. Journal of the Irish Medical Association 1970;63:75-8.
24. Grange JM. Drug-resistance and tuberculosis elimination. Bulletin International Union Against Tuberculosis and Lung Diseases 1990;65:57.
25. Youatt J. A review of the action of isoniazid. American Review of Respiratory Diseases 1969;99:729-49.
26. Shoeb HA, Bowman BU Jr, Ottolenghi AC, Merola AJ. Peroxidase-mediated oxidation of isoniazid. Antimicrob Agents Chemother 1985;27:399-403.
27. Maggliozzo RS, Marcinkeviciene JA. Evidence for isoniazid oxidation by oxypressors mycobaterial catalase-oxidase. Journal American Chemical Society 1996;118:11303-4.
28. Middlebrook G. Isoniazid-resistance and catalase activity of tubercle bacilli. American Review of Tuberculosis 1954;69:471-2.
29. Zhang Y, Heym B, Allen B, Young D, Cole S. The catalase-peroxidase gene and isoniazid resistance of *Mycobacterium tuberculosis*. Nature 1992;358:591-3.
30. Zhang Y, Garbe T, Young D. Transformation with *kat*G restores isoniazid-sensitivity in *Mycobacterium tuberculosis* isolates resistant to a range of drug concentrations. Mol Microbiol 1993;8:521-4.
31. Demple B, Halbrook J. Inducible repair of oxidative DNA damage in *Escherichia coli*. Nature 1983;304:466.
32. Sherman DR, Sabo PJ, Hickey MJ, Arain TM, Mahairas GG, Yuan Y, et al. Disparate responses to oxidative stress in saprophytic and pathogenic mycobacteria. Proc Natl Acad Sci U S A 1995;92:6625-9.
33. Sherman DR, Mdluli K, Hickey MJ, Arain TM, Morris SL, Barry CE III, Stover CK. Compensatory *ahp*C gene expression in isoniazid-resistant *Mycobacterium tuberculosis*. Science 1996;272:1641-3.

34. Chae HZ, Robinson K, Leslie B, Church GB, Storz G, Rhee SG. Cloning and sequencing of thiol-specific antioxidant from mammalian brain, alkyl hydroperoxide reductase and thiolspecific anti-oxidant define a large family of antioxidant enzymes. Proc Natl Acad Sci U S A 1994;91:7017-21.
35. Chae HZ, Chung SJ, Rhee SG. Thioredoxin-dependent-peroxide reductase from yeast. J Biol Chem 1994;269:276-0.
36. Sreevatsan S, Pan X, Zhang Y, Deretic V, Muser JM. Analysis of the *oxy*R-*ahp*C region in isoniazid-resistant and -susceptible *Mycobacterium tuberculosis* complex organims recovered from diseased humans and animals in diverse localities. Antimicrob Agents Chemother 1997;41:600-6.
37. Heym B, Stavropoulos E, Honore N, Domenech P, Saint-Joanis B, Wilson TM, et al. Effects of overexpression of the alkyl hydroperoxide reductase AhpC on the virulence and isoniazid resistance of *Mycobacterium tuberculosis*. Infect Immun 1997;65:1395-401.
38. Altamarino M, Marostenmaki J, Wong A, Fitzgerald M, Black WA, Smith JA. Mutations in the catalase-peroxidase gene from isoniazid-resistant *Mycobacterium tuberculosis* isolates. J Infect Dis 1994;160:1162-5.
39. Stoeckle MY, Guan L, Riegler N, Weitzman I, Kreiswirth B, Kornblum J, et al. Catalase-peroxidase gene sequences in isoniazid-sensitive and -resistant strains of *Mycobacterium tuberculosis* from New York City. J Infect Dis 1993;168:1063-5.
40. Cockerill FR III, Uhi JR, Temesgen Z, Zhang Y, Stockman L, Roberts GD, et al. Rapid identification of a point mutation of the *Mycobacterium tuberculosis* catalase-peroxidase (*kat*G) gene associated with isoniazid resistance. J Infect Dis 1995;171:240-5.
41. Pretorius GS, Van Helden PD, Sergel F, Eisenach KD, Victor TC, et al. Mutations in *kat*G gene sequences in isoniazid-resistant clinical isolates of *Mycobacterium tuberculosis* are rare. Antimicrob Agents Chemother 1995;39:2276-81.
42. Heym B, Alzavi PM, Honore N, Cole ST. Missense mutations in the catalase-peroxidase gene, *kat*G, are associated with isoniazid resistance in *Mycobacterium tuberculosis*. Mol Microbiol 1995;15:235-45.
43. Rouse DA, Devito JA, Li Z, Byer M, Morris SL. Site-directed mutagenesis of the *kat*G gene of *Mycobacterium tuberculosis*: effects on catalase-peroxidase activities and isoniazid resistance. Mol Microbiol 1996;22:583-92.
44. Jaber M, Rattan A, Kumar R. Presence of *kat*G gene in isoniazid-resistant strains of *Mycobacterium tuberculosis*. J Clin Pathol 1996;49:945-6.
45. Kalia A, Ahmad N, Rattan A. Diagnosis of multi-drug resistant tuberculosis: comparison of traditional, radiometric and molecular methods [abstract]. In: Abstracts of the 20th International Congress of Chemotherapy; 29 Jun-3 Jul 1997; Sydney, Australia. Sydney: International Society of Chemotherapy; 1997. p. 211.
46. Heym B, Honore N, Truffot-Pernot C, Banerjee A, Schurra C, Jacobs WR Jr, et al. Implications of multidrug resistance for the future of short-course chemotherapy of tuberculosis: a molecular study. Lancet 1994;344:293-8.
47. Kapur V, Li LL, Hamrick MR, Plikaytis BB, Shinnick TM, Telenti A, et al. Rapid *Mycobacterium* species assignment and unambiguous identification of mutations associated with antibiotic resistance in *Mycobacterium tuberculosis* by automated DNA sequencing. Arch Pathol Lab Med 1995;119:131-8.
48. Morris SL, Bai Gh, Suffys P, Portillo-Gomez L, Fairchok M, Rouse D. Molecular mechanisms of multidrug resistance in clinical isolates of *Mycobacterium tuberculosis*. J Infect Dis 1995;171:954-60.
49. Johnsson K, Froland WA, Schultz PG. Overexpression, purification and characterization of the catalase-peroxidase, katG from *Mycobacterium tuberculosis*. J Biol Chem 1997;272:2834-40.
50. Rouse DA, Li Z, Baig M, Morris SL. Characterization of the *kat*G and *inh*A genes of isoniazid resistant clinical isolates of *Mycobacterium tuberculosis*. Antimicrob Agents Chemother 1995;30:2472-7.
51. Rosner JL. Susceptibility of *oxy*R regulon mutants of *Escherichia coli and Salmonella typhimurium* to isoniazid. Antimicrob Agents Chemother 1993;37:2251-3.
52. Deretic V, Philipp W, Dhandyuthapani S, Mudd MH, Curcic R, Garbe T, et al. *Mycobacterium tuberculosis* is a natural mutant with an inactivated oxidative stress regulatory gene: implications for sensitivity to isoniazid. Mol Microbiol 1995;17:889-900.
53. Wilson TM, Collins DM. *ahp*C, a gene involved in isoniazid resistance of *Mycobacterium tuberculosis* complex. Mol Microbiol 1996;19:1025-34.
54. Banerjee A, Dubnau E, Quemard A, Balasubramanian V, Um KS, Wilson T, et al. *inh*A, a gene encoding a target for isoniazid and ethionamide in *Mycobacterium tuberculosis*. Science 1994;263:227-30.
55. Bergler H, Wallner P, Ebeling A, Leitinger B, Fuchshlschler S, Aschauer H, et al. Protein EnvM is the NADH-dependent-enoyl-ACP-reductase (Fab1) of *Escherichia coli*. J Biol Chem 1994;269:5493-6.
56. Cole ST. *Mycobacterium tuberculosis*: drug-resistance mechanisms. Trends Microbiol 1994;2:411-5.
57. Dessen A, Quemard A, Blanchard JS, Jacobs WR Jr, Sacchettini JC. Crystal structure and function of the isoniazid target of *Mycobacterium tuberculosis*. Science 1995;267:1638-41.
58. Quemard A, Sacchettini JC, Dessen A, Jacobs WR Jr, Blanchard JS, et al. Enzymatic characterization of the target for isoniazid in *Mycobacterium tuberculosis*. Biochemistry 1995;34:8235-41.
59. Johnsson K, King DS, Schultz PG. Studies on the mechanism of action of isoniazid and ethionamide in chemotherapy of tuberculosis. Journal of American Chemical Society 1995;117:5009-10.
60. Takayama K, Schoenes HK, Armstrong EL, Boyle KW. Site of inhibitory action of isoniazid in the synthesis of mycolic acids in *Mycobacterium tuberculosis*. J Lipid Res 1975;16:308-17.
61. Davidson LA, Takayma K. Isoniazid inhibition of the synthesis of mono-saturated long chain fatty acids in *Mycobacterium tuberculosis* H37Ra. Antimicrob Agents Chemother 1979;16:104-5.

62. Mdluli K, Sherman DR, Hickey MJ, Kreiswirth BN, Morris S, Stover CK, Barry LE III, et al. Biochemical and genetic data suggest that *inh*A is not the primary target for activated isoniazid in *Mycobacterium tuberculosis*. J Infect Dis 1996;174:1085-90.
63. Woodley CL, Kilburn JO, David HL, Silcox VA. Susceptibility of mycobacteria to rifampin. Antimicrob Agents Chemother 1972;2:245-9.
64. Ovchinnikov YA, Monastyrskaya GS, Gubanov VV, Lipkin VM, Sverdlov ED, Kiver IF, et al. Primary structure of *Escherichia coli* RNA polymerase nucleotide substitution in the ß-subunit gene of rifampicin resistant *rpo*B255 mutant. Mol Gen Genet 1981;84:536-8.
65. Levin ME, Hatfull GF. *Mycobacterium smegmatis* RNA polymerase: DNA supercoiling, action of rifampicin and mechanism of rifampicin resistance. Mol Microbiol 1993;8:277-85.
66. Ovchinnikov YA, Monastryskaya, GS, Gubanov VV, Lipkin VM, Sverdlov ED, Kiver IF, et al. The primary structure of *Escherichia coli* RNA polymerase. Nucleotide sequence of *rpo*B gene and amino-acid sequence of the ß-subunit. Eur J Biochem 1981;116:621-9.
67. Jin D, Gross C. Mapping and sequencing of mutations in the *Escherichia coli rpo*B gene that leads to rifampicin resistance. J Mol Biol 1988;202:45-58.
68. Telenti A, Imboden P, Marchesi F. Lowrie D, Cole S, Colston MJ, et al. Detection of rifampicin-resistance mutations in *Mycobacterium tuberculosis*. Lancet 1993;341:647-50.
69. Telenti A, Imboden P, Marchesi F, Schidheini T, Bodmer T. Direct, automated detection of rifampicin-resistant *Mycobacterium tuberculosis* by polymerase chain reaction and single-strand conformation polymorphism analysis. Antimicrob Agents Chemother 1993;37:2054-8.
70. Williams DL, Waguespack C, Eisenach K, Crawford JT, Portaels M, Salfinger M, et al. Characterization of rifampicin-resistance in pathogenic mycobacteria. Antimicrob Agents Chemother 1994;38:2380-6.
71. Kapur V, Li LL, Iordanescu S, Hamrick MR, Wanger A, Kreisworth RN, et al. Characterization by automated DNA sequencing of mutations in the gene (*rpo*B) encoding the RNA polymerase ß-subunit in rifampicin-resistant *Mycobacterium tuberculosis* strains from New York City and Texas. J Clin Microbiol 1994;32:1095-8.
72. Felmlee TA, Liu Q, Whelen AC, Williams D, Sommer SS, Persing DH. Genotypic detection of *Mycobacterium tuberculosis* rifampin resistance: comparison of single strand conformation polymorphism and dideoxy fingerprinting. J Clin Microbiol 1995;33:1617-23.
73. Whelen AC, Felmlee TA, Hunt JM, Williams DL, Roberts GD, Stockman L, Persing DH. Direct genotype detection of *Mycobacterium tuberculosis* rifampicin resistance in clinical specimens by using single-tube heminested PCR. J Clin Microbiol 1995;33:556-61.
74. De Benhouwer, Lhiang HZ, Jannes G, Mijis W, Machtelinckx L, Rossau H, et al. Rapid detection of rifampin resistance in sputum and biopsy samples from tuberculosis patients by PCR and line probe assay. Tubercle and Lung Diseases 1995;76:425-30.
75. Cooksey RC, Morlock GP, Glickman S, Crawford JT. Evaluation of a line probe assay kit for characterization of *rpo*B mutations in rifampin resistant *Mycobacterium tuberculosis* isolates from New York City. J Clin Microbiol 1997;35:1281-3.
76. Telenti A, Persing DH. Novel strategies for the detection of drug resistance in *Mycobacterium tuberculosis*. Res Microbiol 1996;147:73-9.
77. Kim BJ, Kim SY, Park B-H, Liu M-A, Park I-K, Bai GH, et al. Mutations in the *rpo*B gene in *Mycobacterium tuberculosis* that ineterfere with PCR-single strand conformation polymorphism analysis for rifampin susceptibility testing. J Clin Microbiol 1997;35:492-4.
78. Thomas JP, Baughan CO, Wilkinson RG, Shephard RG. A new synthetic compound with anti-tuberculous activity in mice: ethambutol (dextro-2,2'-[ethylenediimino]-di-1-butonol). American Review of Respiratory Diseases 1961;83:891-3.
79. Masur H. Recommendations on prophylaxis and therapy for disseminated *Mycobacterium avium* complex disease in patients infected with HIV virus. N Engl J Med 1993;329:828-33.
80. Rastoggi N, Goh KS. Action of 1-isonicotinyl-2-palmitoyl hydrazine against the *Mycobacterium avium* complex and enhancement of its activity by m-flurophenyl alanine. Antimicrob Agents Chemother 1990;34:2061-4.
81. Rastoggi N, Goh KS, Labrausse V. Activity of clathiromycin compared with those of other drugs against *Mycobacterium paratuberculosis* and further enhancement of its extracellular and intracellular activities by ethambutol. Antimicrob Agents Chemother 1992;36:2843-6.
82. Takayama K, Armstrong EL, Kunugi KA, Kilburn JO. Inhibition by ethambutol of mycolic acid transfer into the cell wall of *Mycobacterium smegmatis*. Antimicrob Agents Chemother 1979;16:240-2.
83. Kilburn JO, Takayama K. Effects of ethambutol on accumulation and secretion of trehalose mycolates and free mycolic acid in *Mycobacterium smegmatis*. Antimicrob Agents Chemother 1981;20:401-4.
84. Takayama K, Kilburn JO. Inhibition of synthesis of arabinogalactan by ethambutol in *Mycobacterium smegmatis*. Antimicrob Agents Chemother 1989;33:1493-9.
85. Wolucka BA, McNeil MR, de Hoffman E, Chojnaki T, Brennan PJ. Recognition of the lipid intermediate for arabinogalactan/arabinomanan biosynthesis and its relation to the mode of action ethambutol on mycobacteria. J Biol Chem 1994;269:23328-35.
86. Belanger AE, Besra GS, Ford ME, Mikusova K, Belisle JT, Brennan PJ, Inamine JM. The *emb*AB genes of *Mycobacterium avium* encode an arabinosyl transferase involved in cell wall arabinan biosynthesis that is the target for the antimycobacterial drug ethambutol. Proc Natl Acad Sci U S A 1996;93:11919-24.
87. Telenti A, Philipp WJ, Sreevatsan S, Bernasconi C, Stockbauer KE, Weites B, et al. The *emb* operon, a gene cluster of *Mycobacetrium tuberculosis* involved in resistance to ethambutol. Nat Med 1997;3:567-70.
88. Sreevatsan S, Stockbauer KE, Pan X, Kreisworth BM, Moghazeh SL, Jacobs WR Jr, et al. Ethambutol resistance in *Mycobacterium tuberculosis*: critical role of *emb*B mutations. Antimicrob Agents Chemother 1997;41:1677-81.

89. Konno K, Nagayama H, Oka S. Nicotinamidase in mycobacteria: a method for distinguishing bovine type tubercle bacilli from other mycobacteria. Nature 1959;184:1743-4.
90. Konno K, Feldman FM, McDermot W. Pyrazinamide susceptibility and amidase activity of tubercle bacilli. American Review of Respiratory Diseases 1967;95:461-7.
91. Mackaness GB. The intracellular activation of pyrazinamide and nicotinamide. American Review of Tuberculosis 1953;74:718-28.
92. Scorpio A, Zhang Y. Mutations in *pnc*A, a gene encoding pyrazinamidase/nicotinamidase, cause resistance to the antituberculous drug pyrazinamide in tubercle bacillus. Nat Med 1996;2:662-7.
93. Sreevatsan S, Pan X, Zhang Y, Kreisworth BN, Musser JM. Mutations associated with pyrazinamide resistance in *pnc*A of *Mycobacterium tuberculosis* complex organisms. Antimicrob Agents Chemother 1997;41:636-40.
94. Scorpio A, Lindholm-Levy P, Heifets L, Gilman R, Siddiqi S, Cynamon M, Zhang Y, et al. Characterization of *pnc*A mutations in pyrazinamide resistant *Mycobacterium tuberculosis*. Antimicrob Agents Chemother 1997;41:540-2.
95. Hewlett D, Horn DL, Alfalfa C. Drug resistant tuberculosis: inconsistent results of pyrazinamide susceptibility testing. JAMA 1995;273:916-7.
96. Scorpio A, Collins D, Whipple D, Cave D, Bates J, Zhang Y. Rapid differentiation of bovine and human tubercule bacilli based on a characteristic mutation in the bovine pyrazinamidase gene. J Clin Microbiol 1997;35:106-10.
97. Gay JD, de Young DR, Roberts GD. In vitro activities of norfloxacin and ciprofloxacin against *Mycobacterium tuberculosis, M. avium* complex, *M chelonei, M. forfuitre, and M. kansasii.* Antimicrob Agents Chemother 1984;26:94-6.
98. Gellert M, Mizuuchi K, O'Dea MH, Nash HA. DNA gyrase: an enzyme that introduces superhelical turns into DNA. Proc Natl Acad Sci U S A 1976;73:3872-5.
99. Kirchausen T, Wang JC, Harrison SC. DNA gyrase and its complexes with DNA: direct observation by electron microscopy. Cell 1985;41:933-43.
100. Higgins NP, Peebles CL, Sugino A, Cozzarelli NR. Purification of the subunits of *Escherichia coli* DNA gyrase and reconstitution of enzyme activity. Proc Natl Acad Sci U S A 1978;75:1773-7.
101. Gellart M, O'Dea MH, Itoh T, Tomizava J. Novobiocin and coumeromycin inhibit DNA supercoiling catalyzed by DNA gyrase. Proc Natl Acad Sci U S A 1976;73:4474-8.
102. Sugino A, Peebles CL, Kreuzer KN, Cozzarelli NR. Mechanism of action of Nalidixic acid: purification of *Escherichia coli nal*A gene product and its relationship to DNA gyrase and a novel nicking closing enzyme. Proc Natl Acad Sci U S A 1977;74:4667-71.
103. Kirkegaard K, Wand JC. Mapping the topography of DNA wrapped around gyrase by nucleolytic and chemical probing of complexes of unique DNA sequences. Cell 1981;23:721-9.
104. Shen LL, Pernet AG. Mechanism of inhibition of DNA gyrase by analogues of nalidixic acid: the target of the drugs is the DNA gyrase. Proc Natl Acad Sci U S A 1985;82:307-11.
105. Wilmont CJR, Critchlow SE, Eperon IC, Maxwell A. The complex of DNA gyrase and quinolone drugs with DNA forms a barrier to transcription by RNA polymerase. J Mol Biol 1994;242:351-63.
106. Lewis RJ, Tsai FTF, Wigley DB. Molecular mechanism of drug inhibition by DNA gyrase. Bioessays 1996;18:661-71.
107. Takiff HE, Salazar L, Guerrero C, Philipp W, Huang WM, Kreisworth B, et al. Cloning and nucleotide sequencing of *Mycobacterium tuberculosis gyr*A and *gyr*B genes and detection of quinolone resistant mutations. Antimicrob Agents Chemother 1994;38:773-80.
108. Rees RJ, Maxwell A. DNA gyrase. Structure and function. Crit Rev Biochem Mol Biol 1991;26:335-75.
109. Revel V, Cambau E, Jarlier E, Sougakoff W. Characterization of mutations in *Mycobacterium smegmatis* involved in resistance to fluoroquinolones. Antimicrob Agents Chemother 1994;38:1991-6.
110. Inderlied CB. Antimycobacterial agents: in vitro susceptibility testing, spectrums of activity, mechanisms of action and resistance, and assays for activity in biological fluids. In: Lorain V, editor. Antibiotics in laboratory medicine. Baltimore: Williams and Wilkins, Baltimore; 1991. p. 134-197.
111. Benveinste R, Davies J. Mechanism of antibiotic resistance in bacteria. Annu Rev Biochem 1973;42:471-506.
112. Davies J, Wright JD. Bacterial resistance to aminoglycoside antibiotics. Trends Microbiol 1997;5:234-9.
113. Douglass J, Steyn LM. A ribosomal gene mutation in streptomycin-resistant *Mycobacterium tuberculosis* isolates. J Infect Dis 1993;167:1505-6.
114. Finken M, Kirschner P, Meier, A, Wrede A, Bottger EC. Molecular basis of streptomycin-resistance in *Mycobacterium tuberculosis*: alteration of the ribosomal protein S12 gene and point mutations within a functional 16S rRNA pseudoknot. Mol Microbiol 1993;9:1239-46.
115. Meier A, Kirschner P, Bange FC, Vogel U, Botger EC. Genetic alteration in streptomycin-resistance in *Mycobacterium tuberculosis*: mapping of mutations conferring resistance. Antimicrob Agents Chemother 1994;38:228-33.
116. Nair J, Rouse DA, Bai GH, Morris SL. The *rps*L gene and streptomycin resistance in single and multi-drug resistant strains of *Mycobacterium tuberculosis*. Mol Microbiol 1993;10:521-4.
117. Honore N, Cole ST. Streptomycin resistance in mycobacteria. Anitmicrob Agents Chemother 1994;38:238-42.
118. Woes CR, Gutell RR. Evidence for several higher order structural elements in ribosomal rRNA. Proc Natl Acad Sci U S A 1989;86:3119-22.
119. Shaila MS, Gopinathan RP, Ramakrishnan T. Protein synthesis Mycobacterium tuberculosis H37Rv and the effect of streptomycin in streptomycin susceptible and resistant strains. Antimicrob Agents Chemother 1973;4:205-13.
120. Meier A, Sander P, Schaper KJ, Scholz M, Bottger EC. Correlation of molecular resistance mechanisms and phenotypic resistance levels in streptomycin-resistant *Mycobacterium tuberculosis*. Antimicrob Agents Chemother1996;40:2452-4.
121. Orita M, Suzuki Y, Sekiya T, Hayashi K. Rapid and sensitive detection of point mutations and DNA polymorphisms using the polymerase chain reaction. Genomics 1989;5:875-9.
